# Mechanisms of vascular calcification: cellular phenotype switching drives matrix remodeling and mineralized microenvironment formation

**DOI:** 10.3389/fcvm.2026.1864140

**Published:** 2026-06-18

**Authors:** Haoyu Wen, Weiwei Chen, Yuquan He

**Affiliations:** Department of Cardiology, China-Japan Union Hospital of Jilin University, Changchun, Jilin, China

**Keywords:** epigenetic regulation, matrix remodeling, metabolic disorders, osteogenic differentiation, oxidative stress, vascular calcification, VSMC phenotypic switching

## Abstract

Vascular calcification (VC), a prominent clinical characteristic of cardiovascular diseases, is intricately linked to chronic renal disease, diabetes, atherosclerosis, and other conditions, markedly increasing the risk of cardiovascular events. Traditionally perceived as a passive accumulation due to calcium-phosphate imbalance, recent findings now depict vascular calcification (VC) as an active mineralization process directed by vascular smooth muscle cells (VSMCs). This review seeks to elucidate the crucial role of VSMC phenotypic switching: Under the combined effects of metabolic disturbances (calcium-phosphate dysregulation, glucolipotoxicity), oxidative stress, and chronic inflammation, VSMCs shift from a contractile phenotype to an osteogenic/chondrogenic-like state. This transformation facilitates the establishment of a self-reinforcing mineralized microenvironment through matrix metalloproteinase (MMP)-mediated degradation of the extracellular matrix (ECM), release of matrix vesicles (MVs), and activation of pro-mineralization signal pathways (e.g., Wnt/β-catenin, BMP/SMAD). Additionally, endothelial-mesenchymal transition (EndoMT), macrophage polarization (M1/M2 imbalance), epigenetic regulation (histone modifications, non-coding RNAs), and regulated cell death (apoptosis, pyroptosis, ferroptosis) intensify calcification by releasing mineralization initiators and remodeling the ECM. This review emphasizes the complex interplay between metabolism-related calcification and proposes prospective treatment options, such as targeting metabolic checkpoints (e.g., PDK4, PPARγ), preventing phenotypic flipping, or influencing epigenetic reprogramming. These findings provide a theoretical foundation for the development of targeted therapies in the treatment of vascular calcification.

## Introduction

Vascular calcification (VC), defined by the abnormal accumulation of calcium and phosphate in arterial walls, is a cardiovascular ailment (CVD) closely linked to chronic kidney disease (CKD), atherosclerosis (AS), hypertension, diabetes, and aging et al. It functions as an independent risk factor for significant adverse cardiovascular events (MACE). The incidence of VC has escalated alongside population aging and the increasing incidence of metabolic disorders, resulting in significant socioeconomic and individual health challenges. Uncontrolled calcium accumulation elevates vascular stiffness, reduced compliance, and may lead to luminal narrowing, instigating thrombosis, plaque rupture, myocardial ischemia, left ventricular hypertrophy, and heart failure. The mortality rate due to cardiovascular causes is 3.94 times higher in individuals with calcification compared to those without. This prevalence notably escalates among the elderly, affecting over 90% of males and 67% of females among elderly individuals aged over 80 years (1, 2). The significant morbidity and mortality associated with VC have prompted much investigation into its underlying causes.

Traditional perspectives ascribed vascular calcification (VC) to passive mineral deposition resulting from a calcium-phosphate imbalance. Nonetheless, recent research characterizes it as an active mineralization process facilitated by vascular smooth muscle cells (VSMCs). Key pathological hallmarks include: (1) Vascular smooth muscle cells (VSMCs) phenotypic switching: Transition from a contractile to a synthetic/osteogenic-like phenotype, characterized by the loss of contractile markers [e.g., alpha-smooth muscle actin (α-SMA)] and the activation of osteogenic genes [e.g., runt-related transcription factor 2 (RUNX2), bone morphogenetic protein 2 (BMP2)] (3); (2) Extracellular matrix (ECM) remodeling: Degradation of collagen and elastic fibres, along with the release of matrix vesicles, which facilitate hydroxyapatite nucleation (4); (3) Pro-mineralizing microenvironment: Increased calcium-phosphate levels, secretion of inflammatory cytokines (e.g., TGF-β1, IL-6), and activation of signal pathways such as Wnt/β-catenin (5). This dynamic process highlights vascular calcification (VC) as a pathological cascade driven by multiple synergistic cellular and molecular mechanisms.

This study posits that the phenotypic transformation of vascular smooth muscle cells (VSMCs) serves as the principal driver of vascular calcification (VC), Especially the calcification of the media layer, promoting the remodeling of the extracellular matrix (ECM) and the establishment of pro-mineralizing molecular networks, thereby creating a self-sustaining pathological environment. Key mechanisms include: (1) MMP-mediated ECM degradation: Phenotypically altered VSMCs secrete matrix metalloproteinases (e.g., MMP-3) to degrade collagen and elastin, thereby exposing mineralization nucleation sites and releasing calcium-phosphate-rich matrix vesicles (6); (2) Autocrine signal amplification: Osteogenic-like VSMCs produce TGF-β1 and BMP2, activating SMAD and NF-κB pathways to propagate phenotypic switching in adjacent cells, creating a pathogenic positive feedback loop (5, 7); (3) Matrix stiffness-driven differentiation: Aberrant expression of ECM components (e.g., fibronectin, osteopontin) modifies matrix rigidity, enhancing VSMCs osteogenic differentiation via integrin-FAK signal (8). This framework elucidates the increased vascular calcification susceptibility in chronic kidney disease and diabetes while identifying therapeutic targets for disrupting matrix remodeling or reversing pathological cellular reprogramming.

## Metabolic disorder: a central trigger for cellular phenotypic switching

The pathology of vascular calcification is closely associated with metabolic disorders, wherein imbalances in calcium-phosphorus homeostasis, dysregulated glucose metabolism, and lipotoxicity constitute the principal metabolic triggers for the phenotypic transformation of vascular smooth muscle cells (VSMCs).

These metabolic disorders independently induce reprogramming of the vascular microenvironment (e.g., elevated phosphorus promotes osteogenic differentiation, increased glucose intensifies oxidative damage, and lipid peroxidation activates inflammatory pathways), as well as synergistically (e.g., calcium-phosphorus-glucose metabolism interacts to disrupt pyrophosphate homeostasis, and lipotoxicity exacerbates oxidative stress), culminating in a pro-mineralization vicious cycle. This section will concentrate on three principal metabolic disorders: calcium-phosphorus imbalance associated with chronic kidney disease (CKD), glucose metabolism dysregulation linked to diabetes, and lipid metabolism abnormalities related to atherosclerosis. We will explain how these disorders influence the signal network, change matrix composition, and trigger the inflammation-oxidative stress axis, causing vascular smooth muscle cells to transform into an osteoblast-/chondrocystic-like phenotype, resulting in irreversible vascular calcification.

### Calcium-phosphate imbalance and osteogenic transdifferentiation

The imbalance of calcium and phosphate is a fundamental pathogenic mechanism that contributes to vascular calcification in individuals with chronic kidney disease (CKD). Patients with CKD frequently exhibit hypercalcemia and hyperphosphatemia due to a decreased glomerular filtration rate, impaired vitamin D activation, dysfunction of the calcium-sensing receptor (CaSR), excessive secretion of parathyroid hormone (PTH), and diminished levels of calcification inhibitors, including fetuin-A, matrix Gla protein, and pyrophosphate.

Calcium (Ca^2^⁺) enters vascular smooth muscle cells (VSMCs) through ion channels or calcium-sensing receptors (CaSR), whereas phosphate influx is facilitated by sodium-dependent phosphate transporters 1 and 2 (SPT-1/2) (9). An imbalance of calcium and phosphate, whether separately or synergistically, induces the osteogenic transdifferentiation of vascular smooth muscle cells (VSMCs), marked by the loss of contractile markers (e.g., α-SMA, CNN-1) and the active deposition of hydroxyapatite (10). Calcium-phosphate excess mechanistically activates the Wnt/β-catenin and PI3 K/Akt pathways, leading to the upregulation of osteogenic transcription factors (e.g., Runx2) that facilitate ectopic mineral nucleation (11). The FGF23/klotho axis, a crucial regulator of phosphate balance, has multiple functions: diminished activity facilitates hydroxyapatite crystallization, but prolonged elevation of FGF23 intensifies vascular calcification by stimulating phosphate-induced osteogenesis in vascular smooth muscle cells through ERK1/2 signal (12, 13). These findings suggest that traditional calcification inhibitors may function through diverse mechanisms, underscoring the need for further mechanistic research.

Alongside disorders of calcium and phosphorus metabolism, hyponatremia resulting from an imbalance in water-sodium metabolism and diuretic use can induce intracellular calcium overload by activating the Rac1-Akt signal pathway and down-regulating sodium-calcium exchanger 1 (NCX1) expression; concurrently, it hastens the maturation of extracellular matrix protein modifications (CPPs), establishing a structural foundation for mineral deposition (14). It should be noted that, this regulatory mechanism is still relatively understudied at present, and large-sample clinical cohort studies are further required to verify the clinical correlation between hyponatremia and vascular calcification progression.

Complex bidirectional regulatory systems operate within the network of pathogenic processes to sustain mineralization homeostasis in the body. Recent research indicate that cations like magnesium, zinc, and potassium establish a multilayered network that inhibits calcification by counteracting the deleterious effects of calcium and phosphorus (15, 16), reorganizing cellular signal pathways (17, 18), and preserving vascular matrix integrity (19). Malfunction of these preventive minerals may result in an imbalance between pro- and anti-calcification processes, thereby hastening the vascular calcification process. Magnesium supplementation has demonstrated efficacy in preventing vascular calcification; however, prolonged use may elevate the risk of osteochondrosis by impairing osteoblast function. Consequently, achieving a balance between vascular protection and bone metabolic homeostasis has emerged as a critical challenge for precision intervention (20, 21).

### Matrix-disrupting effects of glucose metabolism dysregulation

Hyperglycemia in diabetes intensifies vascular calcification (VC) via various processes. Chronic hyperglycemia stimulates glycolytic pathways (e.g., hexosamine production), facilitating the buildup of advanced glycation end products (AGEs). Recent evidence designates AGEs as pivotal mediators of diabetic vascular calcification, functioning through both receptor for advanced glycation end products (RAGE)-dependent and RAGE-independent mechanisms to disrupt oxidative stress, inflammation, and mineral deposition (22–25). Hyperglycemia exacerbates vascular damage through distinct molecular mechanisms. Recent research suggests that elevated glucose levels interfere with pyrophosphate metabolism, a critical inhibitor of calcification, thereby increasing the risk of vascular calcification (26). Furthermore, lactate buildup resulting from enhanced glycolysis causes mitochondrial impairment in vascular smooth muscle cells (VSMCs), inhibiting BNIP3-mediated mitophagy and promoting osteogenic phenotypic transition (27, 28). Deviant glucose metabolism further facilitates PDK4-mediated metabolic reprogramming, which inhibits autophagy, amplifies the Warburg effect, and hastens intracellular calcium accumulation (29).

Vascular complications, as a late-stage consequence of diabetes, impose considerable clinical and financial difficulties due to their high frequency and gradual course. Recent preclinical investigations underscore sodium-glucose cotransporter 2 (SGLT2) inhibitors as prospective oral antidiabetic medicines with possible anti-calcification properties; nevertheless, most supporting data come from preclinical models and retrospective observational studies, and there is still a lack of prospective randomized controlled clinical trial evidence to further demonstrate its feasibility (30–33). Clinical findings indicate an inverse relationship between ketone body levels, which rise during glucose deficiency, and the advancement of coronary calcification, suggesting that ketones may serve as protective metabolites. Additional investigation into ketone-mediated processes may provide innovative therapeutic approaches for diabetic vascular problems (34).

### Lipid toxicity cascade in dyslipidemia

Lipid metabolism imbalances induce vascular calcification via a lipotoxic cascade. Clinical observations indicate that hypercholesterolemia and lipoprotein abnormalities (e.g., elevated LDL-C and decreased HDL-C) exhibit a significant positive correlation with the extent of vascular calcification. Various lipid components and metabolites are critical facilitators of the calcification process, influencing key mechanisms such as oxidative stress, inflammatory responses, phenotypic transformation of vascular smooth muscle cells (VSMCs), and remodelling of the extracellular matrix. Consequently, lipid metabolism disorders have emerged as the central pathological nexus linking atherosclerosis and vascular calcification. Oxidized low-density lipoprotein (ox-LDL), a principal pathogenic factor in atherosclerosis, is aberrantly internalized by vascular smooth muscle cells (VSMCs) via scavenger receptors (SR-A/SR-B1), leading to excessive production of reactive oxygen species (ROS) in VSMCs and the activation of the endoplasmic reticulum stress pathway (e.g., the PERK/eIF2α/CHOP signal axis). This process induces the transdifferentiation of VSMCs into osteoblast-like cells and enhances the expression of osteogenic genes such as bone morphogenetic protein-2 (BMP-2) and Runt-related transcription factor 2 (Runx2), thereby accelerating the development of calcified nodules (35). Ox-LDL reduces osteoclast development and functional activity via scavenger receptor A-mediated autophagy and histone K secretion, hence promoting calcification formation (36). Secondly, the aberrant accumulation of free cholesterol and its metabolites within the vascular wall, resulting from dysregulated cholesterol metabolism, induces pyroptosis-like cell death via the activation of NOD-like receptor family pyrin domain containing 3 (NLRP3) inflammasomes. This process culminates in the release of calcium-phosphorus-rich matrix vesicles alongside apoptotic vesicles, which serve as the nucleus for mineralization (37). Lipotoxicity caused by long-chain saturated fatty acids, such as palmitic acid, induces mitochondrial dysfunction in vascular smooth muscle cells (VSMCs), which synergistically enhances osteogenic differentiation in a high-phosphorus milieu by modulating RUNX phosphorylation and the Wnt/β-catenin signal pathway (38, 39). Recent studies have demonstrated that short-chain fatty acids (SCFAs), mainly including acetate, propionate and butyrate, play a pivotal role in the pathogenesis of vascular calcification. Among them, propionate and acetate can markedly ameliorate vascular calcification by mediating gut microbiota remodeling. In contrast, the effect of butyrate exhibits obvious context dependence. In a calcific aortic valve disease model, gut-derived butyrate effectively antagonizes glycolysis-driven calcification by inducing butyrylation of GAPDH at the K263 site and competitively inhibiting lactylation. Nevertheless, in an aortic medial calcification model, butyrate accelerates vascular calcification through dual effects: suppressing the expression of histone deacetylases (HDACs) and activating NF-κB in VSMCs (40–43). Multiple clinical studies have demonstrated the advantageous role of polyunsaturated fatty acids in cardiovascular prevention. Recently, Artiach et al. discovered that specific precursor mediators derived from omega-3 polyunsaturated fatty acids (n-3 PUFA) exhibit deregulated expression in the calcification zone and function as calcification inhibitory molecules, mediating inflammation reduction via the leukotriene E1 and ChemR23 pathways, as evidenced by *in vivo* and *in vitro* studies (44). Conversely, prior research indicated that ω-6 polyunsaturated fatty acids (such as linoleic acid) demonstrate pro-inflammatory properties and may intensify calcification (44). Consequently, additional research is required to explore the molecular mechanisms of various PUFAs subtypes and their ratios in relation to calcification.

In sharp contrast to the anti-mineralization strategies necessary for beneficial outcomes in valvular and mesangial calcification, the primary therapeutic objective in atherosclerosis treatment is to enhance mineralization to stabilize plaques, thereby preventing acute cardiovascular incidents resulting from plaque rupture in highly susceptible intermediate conditions, such as hypo-collagenous and micro-calcified plaques (45, 46). Numerous studies have indicated a positive correlation between statin usage and the advancement of coronary artery calcification. Furthermore, the advantageous effects of statins extend beyond cholesterol reduction; they also prompt macrophages to enhance the secretion of the inflammatory cytokine IL-1β, which incites vascular smooth muscle cells to assume an osteogenic phenotype akin to that of bone (47, 48). The creation of minerals may correlate with the development of more stable plaques, indicating that this treatment may not promptly reverse pre-existing atherosclerosis but instead stabilizes existing plaques (48). While substantial calcifications may temporarily stabilize plaques, ongoing calcification proliferation might result in stenosis, diminished blood flow, and increased systemic resistance (49). The presence of substantial minerals may potentially harden the plaque, rendering interventional methods such as percutaneous transluminal angioplasty (PTCA) and stenting impracticable. This therapeutic conundrum illustrates the “bidirectional regulatory window” inherent in atherosclerotic calcification—moderate mineralization serves as a preventive mechanism for plaque stabilization, while excessive calcification becomes a pathogenic component. The therapeutic application of statins necessitates a balance between immediate advantages and prolonged hazards. Statin intervention is beneficial for stabilizing vulnerable plaques via moderate mineralization in early atherosclerosis, while excessive calcification induced by long-term statin use in advanced atherosclerosis may lead to vascular stenosis and increase the difficulty of interventional therapy, which provides reference for individualized clinical medication decision-making.

Calcium-phosphorus imbalance, aberrant glucose metabolism, and lipotoxicity alter the vascular milieu through synergistic interactions, prompting the transformation of vascular smooth muscle cells (VSMCs) into an osteogenic phenotype and establishing a detrimental cycle of pro-calcification. The mechanisms underlying vascular calcification linked with metabolic disorders are intricate and interrelated. High phosphorus levels degrade PPi by up-regulating tissue non-specific alkaline phosphatase (TNAP), whereas elevated glucose markedly promotes hydroxyapatite nucleation by inhibiting the activity of ENPP1 (pyrophosphate-producing enzyme), resulting in a dysregulated PPi/phosphorus ratio (26, 50). Enhanced glycolysis elevates ATP-associated respiration. This may subsequently prevent calcification in an ENPP1-dependent way, wherein ENPP1 catalyzes the conversion of ATP to pyrophosphate, a powerful calcification inhibitor (51). Moreover, an imbalance of calcium and phosphorus, irregular glucose metabolism, and lipotoxicity might exacerbate vascular calcification by triggering oxidative stress (e.g., reactive oxygen species) or inflammatory responses (NLRP3 inflammasomes). An imbalance of calcium and phosphorus, abnormal glucose metabolism, and lipotoxicity create a mutually reinforcing pathological network through mechanisms including hormonal regulation, inflammatory stress, and reprogramming of energy metabolism, particularly evident in metabolic diseases such as obesity, diabetes, and chronic kidney disease. Interventions aimed at any of these components (e.g., adjusting calcium and phosphorus intake, enhancing insulin sensitivity, or reducing lipotoxicity) may produce combinatorial beneficial effects on the other components.

## Oxidative stress and inflammation: synergistic drivers of phenotypic switching

Metabolic disorders serve as the primary impetus for vascular calcification due to calcium-phosphorus imbalance and glycolipid toxicity. Simultaneously, oxidative stress and chronic inflammation, as synergistic contributors to metabolic diseases, enhance the osteogenic differentiation propensity of vascular smooth muscle cells (VSMCs) by activating the ROS-NF-κB pathway and intensifying the inflammatory cascade. This section will systematically demonstrate how oxidative damage and inflammatory signal, in conjunction with metabolic disorders, create a “bidirectional enhancement loop” by restructuring cellular networks and releasing pro-mineralizing factors that facilitate the irreversible advancement of vascular calcification.

### Oxidative damage reprograms signaling networks

Oxidative stress directly disrupts the homeostasis of vascular smooth muscle cells (VSMCs) by inducing excessive generation of reactive oxygen species (ROS), leading to a transition to an osteogenic/chondroplastic-like phenotype. The accumulation of mitochondrial reactive oxygen species (mtROS) causes DNA damage and triggers the senescence-associated secretory phenotype (SASP), resulting in the aberrant activation of the nuclear factor-κB (NF-κB) and Wnt/β-catenin signal pathways. This promotes the expression of osteogenic differentiation markers (e.g., RUNX2, BMP2), and EVs secreted by stressed VSMCs are rich in alkaline phosphatase and calcium-phosphate complexes, which can directly act as the core scaffold to induce hydroxyapatite nucleation and deposition (52–54). Moreover, oxidative stress intensifies mitochondrial dysfunction and calcification by impairing sirtuin 3 (SIRT3, a mitochondrial deacetylase) activity, resulting in diminished superoxide dismutase (SOD2) function (55). Silencing or inhibiting sirtuin 7 (SIRT7) in vascular smooth muscle cells (VSMC) resulted in heightened intracellular reactive oxygen species (ROS) levels and triggered a Nrf-2-mediated oxidative stress response, while also decelerating VSMC cell cycle progression and expediting cellular senescence, thereby facilitating vascular calcification (56). Reactive oxygen species (ROS) were discovered to augment calcium-dependent signal by activating NADPH oxidases (e.g., Nox5), which subsequently promoted synthetic phenotypic switching and hydroxyapatite deposition in vascular smooth muscle cells (VSMCs) (57, 58).

Mitochondrial damage acts as both a source and a target of oxidative stress, thus forming a vicious cycle. Structural and functional abnormalities of mitochondria lead to massive accumulation of reactive oxygen species and disrupted oxidative stress homeostasis, which further damage mitochondrial DNA and membrane potential, and subsequently activate osteogenic signaling pathways to trigger phenotypic transformation of vascular smooth muscle cells. Excessive mitochondrial fission and downregulated expression of fusion-related proteins mitofusin 1/2 (MFN1/2) and optical atrophy 1 (Opa1) induce disordered mitochondrial dynamics, exacerbating mitochondrial fragmentation and energy synthesis failure. Damaged mitochondria cannot be timely eliminated due to impaired mitophagy, continuously releasing oxidative stress and pro-calcific signals, and further facilitating calcification by inducing cellular senescence and inflammatory microenvironment. Moreover, inhibited mitochondrial oxidative phosphorylation reduces ATP production and shifts cellular metabolism toward glycolytic reprogramming. Combined with intracellular calcium overload caused by disturbed mitochondrial calcium transport, calcium-phosphate crystal deposition is more likely to occur under hyperphosphatemic conditions. Abnormal opening of mitochondrial permeability transition pores mediates VSMC apoptosis, and apoptotic bodies serve as scaffolds for calcified crystal deposition. Impaired sirtuin 1 (SIRT1)/peroxisome proliferator-activated receptor gamma coactivator 1-alpha (PGC-1α) signaling hampers mitochondrial biogenesis and fails to compensate for mitochondrial injury. Collectively, these alterations involving oxidative stress, dynamic imbalance, defective mitophagy, disturbed energy metabolism, dysregulated calcium-phosphate homeostasis, apoptosis and senescence synergistically promote the initiation and progression of vascular calcification (59).

Related treatment investigations shown that mitochondria-targeted antioxidants (e.g., MitoQ) markedly postponed vascular calcification by diminishing mtROS and apoptosis (54). Apelin-13 may mitigate high glucose-induced calcification in MOVAS cells by decreasing ROS-mediated DNA damage and regulating MAPK and PI3 K/AKT pathways (60). Celastrol alleviates vascular calcification via anti-oxidative stress effects. SGLT2 inhibitors block the ATF6-dependent endoplasmic reticulum stress pathway. Additionally, mitochondrion-targeted nanodrugs delivering natural antioxidants suppress calcification progression by restoring mitochondrial function and eliminating mitochondrial reactive oxygen species (mtROS) (61, 62). The modulation of the oxidative damage signal network is pivotal for intervention; thus, antioxidants may represent a novel therapeutic option. Reducing the production of oxidative stress products or diminishing the degradation of antioxidants could significantly lower the incidence of vascular calcification.

### Inflammatory cascades amplify pro-mineralization signals

Chronic inflammation and oxidative stress create a positive feedback loop that together promotes vascular calcification. Inflammatory stimuli [e.g., interleukin-6 (IL-6), tumor necrosis factor-alpha (TNF-α)] enhance the expression of pro-calcification genes [e.g., alkaline phosphatase (ALP), osteopontin (OPN)] and suppress the expression of calcification inhibitors [e.g., matrix Gla protein (MGP)] via the activation of NF-κB and NLRP3 inflammatory vesicles (63, 64). Macrophages serve as the primary effector cells in the inflammatory response and facilitate the osteogenic transdifferentiation of vascular smooth muscle cells (VSMCs) by secreting inflammatory mediators such as IL-6 and TNF-α (65). Exosomes (EVs) released by macrophages transport pro-inflammatory miRNAs (e.g., miR-155) and calcification-related proteins, which directly promote osteogenic phenotypic transdifferentiation and extracellular matrix (ECM) mineralization in vascular smooth muscle cells (VSMCs) (66, 67). Component 5a (C5a), a cleavage product of C5 in the complement system, is the most potent pro-inflammatory cytokine. It induces endoplasmic reticulum (ER) stress through the C5a-C5aR1 interaction, subsequently activating the PERK-eIF2α-ATF4 pathway, which accelerates the osteogenic transdifferentiation of vascular smooth muscle cells (VSMCs) (68). Chronic inflammation results in increased reactive oxygen species (ROS), which stimulates the p38 mitogen-activated protein kinase (MAPK) pathway and induces the activation of RAGE receptors in vascular smooth muscle cells (VSMCs), establishing a ROS-RAGE positive feedback loop. This mechanism is especially evident in models of high-dose erythropoietin (EPO)-induced vascular calcification, wherein calcium salt deposition escalates by 40%-60% in inflammatory settings (69). Conversely, antioxidants (e.g., Celastrol) impede both reactive oxygen species (ROS) and inflammatory mediators (e.g., IL-1β), disrupting this detrimental cycle (70). Hydrogen sulphide (H2S) creates a molecular link between inflammation and mineralization by modulating NF-κB signal, hence uncovering a synergistic mechanism between the two (64, 71).

## Cellular phenotypic switching: executors of matrix remodeling

Metabolic disorder-induced oxidative stress outburst and chronic inflammatory cascade can crosstalk through multiple intracellular signaling pathways, further triggering and initiating the phenotypic reprogramming of vascular wall cells. These events act as crucial upstream initiating signals that drive the pathological phenotypic switching of vascular smooth muscle cells, endothelial cells, and macrophages. In this context, osteogenic transdifferentiation of VSMCs, endothelial-to-mesenchymal transition (EndoMT), and macrophage polarization collectively function as the core executors of matrix remodeling. This section focuses on the molecular mechanisms underlying these cellular behaviors, and elucidates how they secrete pro-mineralization factors, degrade ECM components and release matrix vesicles, thereby translating pathological signals into the substantive construction of a pro-calcifying microenvironment.

### Osteogenic transdifferentiation of smooth muscle cells

The osteogenic-like transdifferentiation of vascular smooth muscle cells (VSMCs) is a primary pathogenic process underlying vascular calcification. In the phenotypic transition, the contractile markers of vascular smooth muscle cells [α-SMA, smooth muscle protein 22-alpha (SM22α)] are markedly downregulated, while the osteogenic transcriptional regulatory network is activated, with key components RUNX2, msh homeobox 2 (MSX2), SRY-box 9 (SOX9), osterix (OSX) collaboratively influencing cell destiny remodeling (72, 73). Among them, RUNX2 acts as a core transcription factor and plays a pivotal hub role: it is not only an essential regulatory factor indispensable for bone formation, but also significantly upregulated and stabilized under conditions such as hyperphosphatemia, oxidative stress, and diabetes, thereby directly driving the expression of calcification-related genes (183). At the molecular level, MSX2 promotes the expression of RUNX2 and OSX by initiating a transcriptional cascade, with OSX activation reliant on an RUNX2-mediated positive feedback loop (74, 75). Concurrently, SOX9 interacts with RUNX2, inhibiting contractile phenotype genes while simultaneously activating osteogenic differentiation genes via a dual regulatory mechanism (4, 76). In addition, this transcriptional network is finely modulated by multiple post-translational modification mechanisms. For instance, CK2 (casein kinase 2)-mediated phosphorylation of RUNX2 recruits the deubiquitinated-USP7 (ubiquitin-specific protease 7) to enhance RUNX2 protein stability, thereby facilitating osteogenic differentiation and improving bone metabolism (77). Meanwhile, p300-mediated RUNX2 acetylation not only stabilizes its protein structure but also markedly elevates its transcriptional activity, driving the expression of bone remodeling genes such as MMP-13 (78). Moreover, CTCF synergizes with H3K27ac to upregulate P300 expression, which further amplifies the acetylation effect of RUNX2 and strengthens the osteogenic/chondrogenic differentiation program (79). The expression of MSX2 is regulated by the β-catenin/TCF signaling pathway, while XBP1u indirectly inhibits MSX2 transcription by promoting the ubiquitination and degradation of β-catenin, indicating an indirect modulation of MSX2 activity by the modification network (80).The aforementioned transcription factors facilitate the secretion of mineralization-promoting factors, including alkaline phosphatase (ALP), collagen type I (Col Ⅰ), and bone morphogenetic protein 2 (BMP2) by vascular smooth muscle cells (VSMCs) via the modulation of downstream effector molecules, thereby expediting the mineralization process through the reorganization of enzymatic reactions and signal pathways (74). ALP, a crucial functional molecule, is directly regulated by RUNX2, and its increased activity can disrupt calcium-phosphorus equilibrium and facilitate hydroxyapatite nucleation by hydrolyzing pyrophosphate (PPi) to produce inorganic phosphate (Pi) (81). BMP2, a member of the transforming growth factor β (TGF-β) family, enhances osteogenic-like transdifferentiation signals by promoting the expression of molecules including MSX2, RUNX2, and lipoprotein receptors; its specific inhibition markedly mitigates the advancement of vascular calcification (81). Metabolic disorders, such as calcium-phosphorus imbalance and glucose-lipid metabolism disorders, serve as primary catalysts for the phenotypic transformation of vascular smooth muscle cells. They facilitate the phosphorylation of RUNX2 within the nucleus, thereby initiating the transcription of osteogenic genes via the activation of the Wnt/β-catenin, PI3 K/Akt, and extracellular signal-regulated kinase 1/2 (ERK1/2) signal pathways. This transformation is accompanied by a morphological change from a long spindle shape to a polygonal shape, as well as the release of stromal vesicles from vascular smooth muscle cells. These alkaline phosphatase-rich vesicles enhance calcium salt deposition through localized phosphatase activity (82). Simultaneously, metabolic problems might intensify the osteoblastic differentiation of vascular smooth muscle cells by provoking oxidative stress and inflammatory responses. Deficient mitochondrial autophagy has been shown to aggravate osteogenic-like differentiation of vascular smooth muscle cells, whereas GLP-1 receptor agonists (e.g., Exendin-4) impede phenotypic flipping by promoting mitochondrial autophagy (83). Moreover, the bone morphogenetic protein (BMP) family, such as BMP2, along with non-coding RNAs such circACTA2, enhances the mineralization propensity of vascular smooth muscle cells (VSMCs) by modulating NLRP3 inflammasome activity (84).

### Endothelial dysfunction and EndoMT

Endothelial mesenchymal transition (EndoMT) is a significant contributor to the establishment of the vascular calcification milieu. In their normal state, vascular endothelial cells are crucial for vascular homeostasis by preserving barrier function, regulating vascular tone, and inhibiting aberrant inflammatory responses (85). Pathological stimuli, including metabolic disorders, oxidative stress (e.g., ox-LDL accumulation), inflammatory factors (e.g., TNF-α, IL-6), and abnormal mechanical stress, activate signal pathways such as TGF-β/Smad, Notch, and Wnt/β-catenin. Consequently, endothelial cells undergo phenotypic transformation, progressively losing typical endothelial characteristics (e.g., downregulation of vascular endothelial cadherin and cluster of differentiation 31) and acquiring mesenchymal traits (e.g., upregulation of α-SMA, Vimentin, and fibronectin). This transformation enhances the plasticity of endothelial cells, allowing them to differentiate into osteoblasts or chondrocyte-like cells, which are directly implicated in vascular calcification (86). Concurrently, EndoMT conveys osteogenic signals to vascular smooth muscle cells through the release of extracellular vesicles (EVs), facilitating the conversion of vascular smooth muscle cells to an osteogenic-like phenotype, so establishing a detrimental cycle (87). Exosomes derived from endothelial cells, stimulated by a high-phosphorus milieu, promote the calcification of vascular smooth muscle cells (VSMCs) through the Wnt/β-catenin pathway (88). Additionally, the cyclin-dependent kinase 1 (CDK1)/ETS variant 2 (ETV2) axis serves as a crucial regulator of the transition of endothelial cells to osteoblast-like cells; the inhibition of CDK1 obstructs the endothelial-to-mesenchymal transition (EndoMT) and diminishes calcification (89). The pro-inflammatory cytokines TNF-α and IL-1β initiate EndoMT in human aortic endothelial cells, thereby enhancing their susceptibility to BMP-9-induced osteogenic differentiation and thereby facilitating calcification development (90). Some endothelial cells display an intermediate phenotype (partial EndoMT) possessing both endothelial and mesenchymal characteristics, and this dynamic, reversible condition may continuously enhance local inflammatory signals via paracrine cytokines such as IL-6 and TGF-β (91). Concurrently, metabolic reprogramming (e.g., augmented glycolysis) further strengthens the process of EndoMT via epigenetic regulation (e.g., histone acetylation) (92, 93). Emerging studies have demonstrated that arterial bifurcations, especially under hypertensive conditions, are prone to generating oscillatory shear stress (OSS) and turbulent flow due to unique hemodynamic characteristics, which readily induce endothelial-to-mesenchymal transition (EndoMT) and subsequent vascular calcification (94). Distinct from the unidirectional laminar flow in straight arterial segments, such flow patterns render arterial bifurcations the predominant anatomical sites for atherosclerotic plaque deposition. Mechanistically, endothelial cells (ECs) can sense blood flow and respond to mechanical signals derived from different flow patterns: laminar flow inhibits vascular calcification by suppressing the endothelial BMP/SMAD1/5 signaling pathway in a Krüppel-like factor 2 (KLF2)-dependent manner; whereas turbulent flow triggers EndoMT and vascular calcification via downregulation of KLF2 (95). Furthermore, the removal of the basement membrane protein nidogen-2 impairs interactions between endothelial and smooth muscle cells, intensifying extracellular matrix remodeling and calcification, indicating that the preservation of endothelial microenvironmental homeostasis is essential for preventing pathological mineralization (96).

Therapeutic methods targeting the suppression of EndoMT have demonstrated efficacy in decelerating vascular calcification. SB216763, a glycogen synthase kinase 3β (GSK3β) inhibitor, stabilizes β-catenin and promotes osteoblast-like cell development towards the endothelial lineage, therefore diminishing calcification (91, 97). Cinacalcet mitigates aortic calcification in a uremic model through the inhibition of EndoMT (98).

### Macrophage polarization and osteoclast-like differentiation

Macrophage polarization status directly influences the progression and patterns of vascular calcification. As key immune cells dynamically responding to and actively regulating the calcification process, macrophages participate profoundly in extracellular matrix remodeling and the construction of the mineralizing microenvironment through phenotypic polarization and functional transitions. Traditional physiological and pathological studies have long interpreted macrophage functions based on the classical M1/M2 binary polarization framework. Under physiological conditions, macrophages maintain a dynamically balanced polarization phenotype. Classically activated M1 macrophages are predominantly pro-inflammatory and highly express TNF-α, IL-6, iNOS and other mediators, whereas alternatively activated M2 macrophages exert anti-inflammatory and tissue repair functions by secreting IL-10, TGF-β, and arginase-1. However, under pathological stimuli such as hyperlipidemia, hyperphosphatemia, and advanced glycation end products, macrophage polarization shifts toward a pro-inflammatory and pro-fibrotic M1-dominant phenotype, disrupting the homeostatic balance of local mineralization regulation.

In recent years, single-cell transcriptomic studies have broken through the limitations of the traditional paradigm and confirmed that macrophages within calcified vessel walls and atherosclerotic plaques exhibit profound phenotypic heterogeneity. A large number of intermediate states and functionally specific subpopulations cannot be classified by the conventional M1/M2 dichotomy. The binary classification only roughly summarizes the overall functional tendency of macrophages and fails to truly reflect the continuum and functional diversity of macrophage lineages in the calcified micro-environment. Among them, characteristic SPP1⁺ and LYVE-1⁺ macrophage subpopulations have been specifically identified in calcified lesions and do not conform to the classical M1/M2 spectrum. The pro-inflammatory SPP1⁺ macrophage subset acts as a pivotal functional population in the calcified microenvironment. By secreting inflammatory mediators such as TNF-α, releasing calcium-phosphorus-containing apoptotic bodies, and discharging pro-mineralization extracellular vesicles, SPP1⁺ macrophages directly trigger osteogenic transdifferentiation of VSMCs and initiate microcalcification formation, serving as a critical cellular source driving ectopic vascular mineralization. The LYVE-1⁺CCL24⁺ macrophage subpopulation further aggravates mineral deposition by paracrine secretion of chemokines including CCL24, which induces the transformation of VSMCs into osteogenic/chondrocyte-like cells (67).

Meanwhile, within the calcified microenvironment, macrophages can differentiate into an osteoclast-like phenotype. This process resembles osteoclast formation in bone metabolism and is driven by signals such as RANKL and M-CSF. Nevertheless, the role of macrophage osteoclast-like transformation in vascular calcification is not unidirectional. Early microcalcification is triggered by macrophage-mediated inflammation, and osteoclast-like macrophages may participate in the clearance or remodeling of calcified lesions by expressing bone resorption-related genes. By contrast, osteoclast-like macrophages surrounding macro-calcified lesions highly express the osteoclast-associated gene TRAP, secrete matrix metalloproteinases, and engage in collagen remodeling. These effects facilitate the replacement of the lipid necrotic core by calcified matrix, thereby exhibiting osteoid metaplasia characteristics analogous to those in bone metabolism (67, 99, 100). In summary, macrophage osteoclast-like differentiation exhibits a high degree of context dependence in vascular calcification: it not only represents a consequence of inflammation-driven microcalcification, but also manifests as a reparative phenotype within the late calcified microenvironment, while forming a positive feedback loop with the osteogenic transdifferentiation of VSMCs.

Notably, macrophages finely modulate the mineralization balance via dual mechanisms. On the one hand, they inhibit mineralization by upregulating pyrophosphate metabolism or secreting factors such as CCN3. On the other hand, under metabolic disorders such as diabetes, conventionally anti-inflammatory M2 macrophages may acquire pro-calcific properties due to miR-32 overexpression or metabolic reprogramming. In addition, dynamic switching of macrophage phenotypes mediated by signaling pathways including NF-κB and YAP-TEAD not only alters the calcification potential of extracellular vesicles but also remodels the extracellular matrix microenvironment by regulating TNAP activity, highlighting their pivotal role as core executors of matrix remodeling throughout multistage vascular calcification (101).

To date, targeted modulation of macrophage polarization and the balance of specific subpopulations has emerged as a novel strategy for intervening vascular calcification. Hydrogen sulfide promotes macrophage polarization toward a reparative phenotype via the STAT6/PPARγ pathway, suppresses the activation of pro-calcific subpopulations such as SPP1⁺, and simultaneously inhibits VSMC proliferation and excessive ECM deposition, thereby exerting multiple anti-calcific effects. PPARγ agonists facilitate polarization toward a protective macrophage phenotype and inhibit NLRP3 inflammasome activity. In contrast, blockade of TLR4 signaling or STAT1 knockdown effectively alleviates the pro-calcific effects of macrophages induced by ox-LDL (102–105).

### Distinct contributions of cell phenotype switching to different vascular calcification subtypes

Vascular calcification can be classified into medial calcification and intimal calcification. Under different pathological backgrounds such as chronic kidney disease, atherosclerosis, and diabetes, the involvement degrees of VSMC osteogenic transdifferentiation, EndoMT, and macrophage polarization vary. Nevertheless, VSMC phenotypic switching always acts as the core hub and ultimate effector in the initiation and progression of all types of calcification. Medial calcification associated with chronic kidney disease is mainly induced by hyperphosphatemia and mineral metabolic disorders, which directly and preferentially drive the phenotypic transition of medial VSMCs into an osteoblast-like phenotype. By activating signaling pathways including Wnt/β-catenin and BMP, remodeling the extracellular matrix and releasing matrix vesicles, VSMCs dominate the initiation and progression of calcification, while EndoMT and macrophage polarization only exert secondary synergistic and amplifying effects. In atherosclerosis-related intimal calcification, lipid deposition, inflammation and disturbed blood flow tend to initially induce endothelial dysfunction and EndoMT, and simultaneously promote macrophage polarization to form specific subpopulations such as SPP1⁺ and LYVE-1⁺. These cells construct a pro-calcification microenvironment via inflammatory cytokines and exosomes. However, the above upstream signals ultimately act on VSMCs and induce their osteogenic phenotypic switching to complete the formation and expansion of micro-calcified lesions. Diabetes-associated vascular calcification presents combined pathological features of both medial and intimal lesions. Glucose and lipid metabolic disorders can not only directly trigger VSMC osteogenic transdifferentiation, but also amplify pathological effects by facilitating EndoMT and shifting macrophages toward a pro-inflammatory phenotype, with VSMCs remaining the core node of multicellular crosstalk. Overall, although the activation sequence and participation intensity of the three cell types differ across vascular beds and disease backgrounds, all cases follow a general rule: VSMC phenotypic switching plays a dominant role, while EndoMT and macrophage polarization serve as upstream synergistic amplifiers. This provides a theoretical basis for differentiated targeted intervention against distinct subtypes of vascular calcification.

## Epigenetics and cell death: deep regulatory layers of matrix remodeling

Cellular phenotypic transitions lay the structural foundation for vascular calcification by directly remodeling matrix components and signaling microenvironments. However, the profound regulation of this process relies on the dynamic reprogramming of gene expression by epigenetic modifications and the release of mineralization initiators by cell death. In the following section, we will resolve how epigenetic mechanisms such as histone acetylation and non-coding RNA regulation stabilize the osteogenic phenotype, and explore how death modes such as apoptosis and pyroptosis synergistically advance irreversible mineralization of the ECM through calcium and phosphorus efflux and pro-inflammatory factor release.

### Epigenetic reprogramming of phenotypic switching

Phenotypic switching in vascular smooth muscle cells (VSMC) is a fundamental mechanism of vascular calcification, with epigenetic changes serving a crucial regulatory function in this process. Histone modifications (e.g., acetylation, methylation, lactylation) and DNA methylation facilitate the transition of vascular smooth muscle cells (VSMCs) from a contractile phenotype to an osteogenic/calcifying phenotype by altering chromatin structure or modulating gene expression. For instance, the downregulation of SIRT1 (sirtuin 1), a NAD⁺-dependent histone deacetylase, results in elevated histone acetylation, activation of osteogenesis-related genes (e.g., Runx2, BMP2), and enhancement of calcification (56, 106, 107). Likewise, abnormal regulation of histone methyltransferases (e.g., H3K9me3) can also reinforce the osteogenic phenotype of vascular smooth muscle cells by augmenting methylation alterations in the promoter regions of osteogenic genes (108, 109). The orphan nuclear receptor NR4A3 modulates the expression of the downstream gene Phospho1, which facilitates calcification via its phosphatase activity, by enhancing the lactylation of histone H3K18la (28). In addition, recent studies have revealed that N6-methyladenosine (m6A) modification exerts a target-dependent dual regulatory effect on vascular calcification (VC). In chronic kidney disease-associated vascular calcification, METTL3-mediated m6A modification inhibits the osteogenic transdifferentiation of VSMCs by regulating target genes such as Runx2, and METTL3 deficiency conversely exacerbates calcification (110, 111). In calcific aortic valve disease, downregulated ALKBH5 expression leads to elevated m6A levels and subsequently promotes the calcification of valvular interstitial cells. Moreover, m6A modification-mediated degradation of IRAK-M also potentiates the inflammatory response and calcification progression (112, 113). Long chain non-coding RNAs, such as H19, have been shown to engage with chromatin remodeling complexes via epigenetic mechanisms, including DNA methylation, to suppress the expression of phenotypic markers associated with vascular smooth muscle cell contraction, such as SM22α, thereby facilitating vascular calcification (107, 114). Circular RNA molecules (e.g., circ_0008362) are abundant in endothelial cell-derived extracellular vesicles and function as adsorbent sponges for small RNA molecules, hence facilitating vascular calcification, such as by sequestering miR-1251-5p to enhance Runx2 expression (115, 116). Collectively, these epigenetic reprogramming events form a molecular “switch” for vascular calcification, offering a novel avenue for targeted intervention.

### Cell death-derived mineralization initiators

Cell death is both a result of vascular calcification and an active mechanism that facilitates the development of a mineralized stromal milieu. Factors initiating mineralization, released by various modes of cell death, alter the microenvironment through multiple mechanisms: matrix vesicles, apoptotic vesicles, and necrotic debris serve as mineralization “seeds” and scaffolds; concurrently, increased concentrations of calcium and phosphorus ions, the degradation of anti-mineralization proteins, and the activation of pro-mineralization signals (e.g., BMP-2, Wnt/β-catenin) collectively foster a localized microenvironment conducive to mineralization. For instance, persistent endoplasmic reticulum stress in vascular smooth muscle cells excessively activates three core pathways including PERK, IRE1α and ATF6, thereby triggering the RIPK1/RIPK3/MLKL-mediated necroptosis pathway. Matrix vesicles released by apoptotic VSMCs, such as Ost-MVs and VSMC-MVs, are rich in calcium, phosphorus and alkaline phosphatase. These vesicles can deposit directly into the extracellular matrix (ECM) and accelerate vascular calcification via the Ras-Raf-ERK signaling pathway (117, 118). Matrix vesicles produced during the apoptosis of vascular smooth muscle cells contain essential enzymes for mineralization, such as alkaline phosphatase (ALP), and the polarity reversal of phospholipids on their membrane surface enhances their capacity to bind calcium ions, facilitating the formation of initial mineralization sites (119). Histones (e.g., H3K9la, H3K14la) released from necrotic cells activate osteogenic genes (e.g., Runx2, BMP2) via lactation modification and facilitate aberrant remodeling of collagen I, creating a pro-mineralization milieu (120, 121). Moreover, endoplasmic reticulum stress modulates the calcium transport capacity of apoptotic vesicles via the IRE1-XBP1 pathway, while the disruption of membrane integrity resulting from compromised mitochondrial autophagy releases antioxidant enzymes, including superoxide dismutase 2 (SOD2), thereby intensifying the local oxidative microenvironment and facilitating calcium salt deposition (122). Scorched death, classified as inflammatory cell death, is marked by GSDMD pore proteins that facilitate cell membrane perforation, resulting in unregulated calcium influx from intracellular calcium stores (such as the endoplasmic reticulum) and exacerbating the imbalance of extracellular calcium and phosphorus ion concentrations (123–125). Concurrently, reactive oxygen species (ROS) and proteases, such as caspase-1, released by pyroptotic cells, destroy anti-mineralization proteins, including fetuin-A and osteoprotegerin (OPG), within the matrix, thereby compromising the mineralization inhibitory barrier of the matrix (124, 126). Recent hotspot studies indicate that iron and copper overload catalyze the calcification process by instigating specific cell death mechanisms (e.g., ferroptosis, cuproptosis). Iron ions promote lipid peroxidation through the Fenton reaction, resulting in the inactivation of glutathione peroxidase 4 (GPX4) and the compromise of membrane integrity, alongside the release of mineralized vesicles rich in iron-phosphorus complexes (127). Conversely, copper ions facilitate the degradation of vascular matrix integrity by activating the NLRP3 inflammasome, leading to localized cell death, disruption of vascular matrix integrity, and the induction of oxidative stress that releases pro-calcification factors (128). These variables create a positive feedback loop with ECM remodeling (e.g., increased collagen cross-linking, degradation of elastic fibers), ultimately resulting in an irreversible pathological process of vascular calcification. Notably, the pathological relevance and clinical significance of ferroptosis and cuproptosis in human vascular calcification remain to be further validated by high-quality clinical studies. In summary, cell death is not solely a consequence of pathological injury, but also a crucial component of the active release of mineralization-inducing chemicals that facilitate matrix remodeling. By modulating calcium and phosphorus ion balance, matrix protein composition, and the inflammatory microenvironment, it transforms cellular damage into ectopic mineralization at the tissue level. A comprehensive examination of the molecular mechanisms underlying various modes of cell death and their interplay with phenotypic transformation could yield novel insights for the formulation of anti-calcification therapies aimed at inhibiting pathological cell death or obstructing the release of mineralization initiating factors.

## Extracellular matrix remodeling: final assembly of the mineralized microenvironment

**Figure 1:**
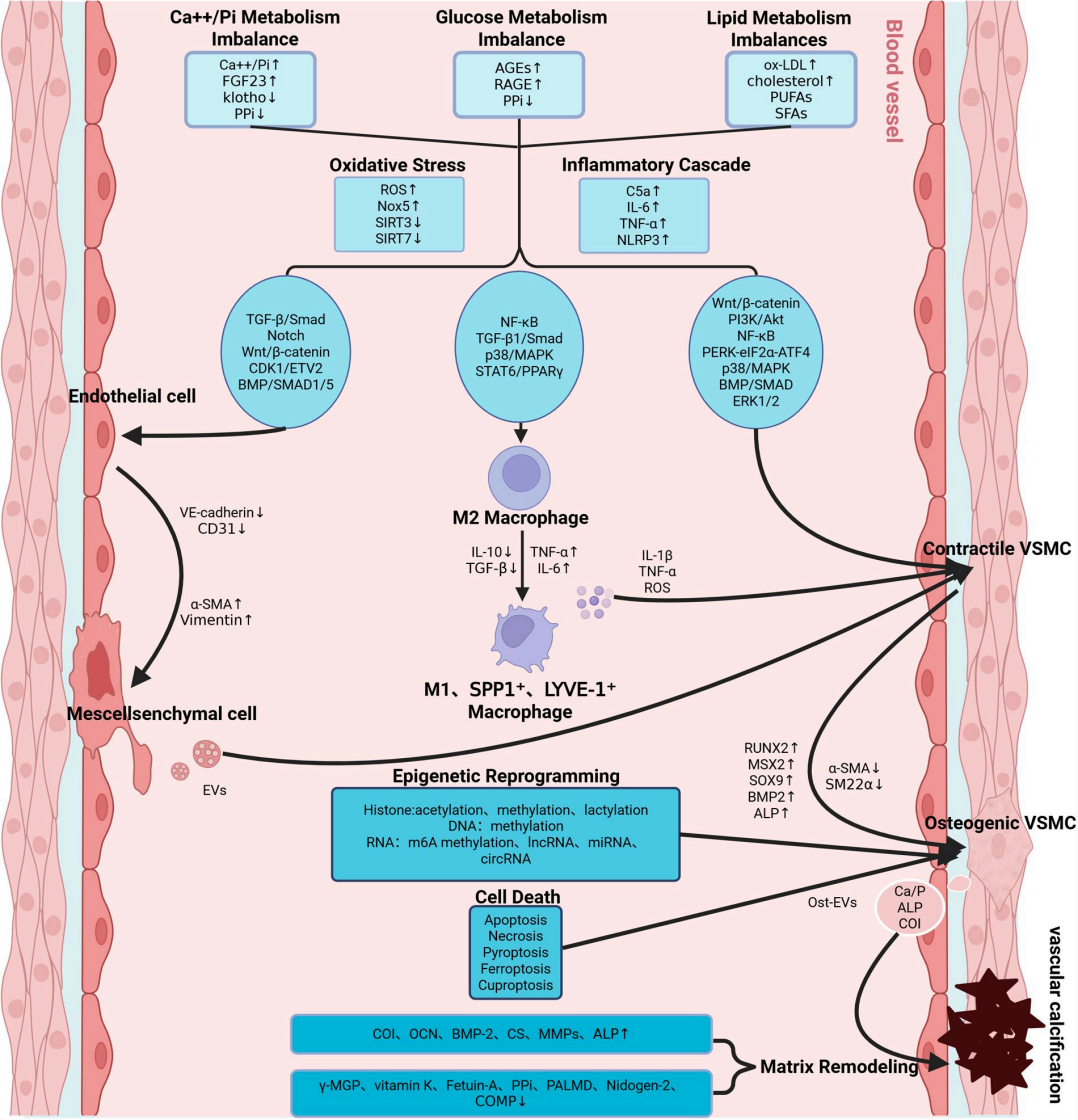
Schematic diagram of the molecular mechanism network underlying vascular calcification induced by multiple pathogenic factors.

The aforementioned pathological events, including cellular phenotypic switching, oxidative stress, chronic inflammation, epigenetic aberrations and programmed cell death, ultimately converge on the vascular extracellular matrix (ECM), forming a complex regulatory network of vascular calcification (Figure 1). Remodeling of ECM structure and function drives the establishment of a pathological mineralized microenvironment. The vascular ECM not only provides structural support for blood vessels but also serves as a pivotal platform for regulating mineral homeostasis, mediating signal transduction and initiating mineral nucleation. Its homeostatic imbalance is mainly manifested as dysregulation of the endogenous mineral absorption network, functional impairment of endogenous anti-mineralization factors, abnormal extracellular vesicle-mediated mineral nucleation, and excessive accumulation of pro-mineralization matrix components. This section systematically elaborates the specific mechanisms by which ECM remodeling contributes to the progression of vascular calcification from the above four perspectives.

### Dysregulation of endogenous mineral absorption network

The endogenous mineral absorption network mainly involves intestinal calcium and phosphorus uptake, renal reabsorption and excretion of minerals, as well as the delicate homeostasis of circulating minerals. These processes are profoundly dysregulated in the context of chronic kidney disease (CKD), acting as a core driver of the initiation and progression of vascular calcification (VC). Under physiological conditions, intestinal phosphate absorption is mediated by sodium-dependent phosphate cotransporters, while the kidney maintains serum phosphate homeostasis via excretion regulation. Vascular smooth muscle cells (VSMCs) sense extracellular calcium levels through calcium-sensing receptor (CaSR) to sustain intracellular calcium homeostasis, and sarco/endoplasmic reticulum Ca^2^⁺-ATPase 2 (SERCA2) ensures normal calcium signal transduction by modulating endoplasmic reticulum calcium uptake. During the progression of CKD-mineral and bone disorder (CKD-MBD), renal insufficiency leads to impaired phosphate excretion and subsequent hyperphosphatemia. The resultant systemic mineral homeostasis disruption directly triggers osteogenic/chondrogenic transdifferentiation of VSMCs, thereby initiating pathological calcium-phosphate deposition. Notably, vascular calcified tissues are not merely passive deposition sites. Accumulating evidence demonstrates that calcified arteries can actively take up acutely fluctuating circulating phosphate, with the uptake magnitude positively correlated with existing calcification burden. This indicates that calcified blood vessels act as a participatory organ in mineral homeostasis, further disturbing systemic phosphate distribution and forming a self-perpetuating vicious cycle (129). At the molecular level, irreversible oxidation of the critical cysteine site C674 in SERCA2 induces functional impairment, disrupts intracellular calcium homeostasis, exacerbates calcium signaling disturbance, and ultimately accelerates mineralization (130). Meanwhile, reduced *α*-Klotho expression abrogates its protective effect against vascular aging, which is strongly correlated with the severity of aortic calcification and adverse clinical outcomes (131). From a therapeutic perspective, calcimimetics exert anti-calcification effects by activating residual CaSR activity (132). Although vitamin K-dependent proteins possess inherent anti-calcification potential, vitamin K supplementation yields limited clinical benefits in CKD patients, implying the existence of complex compensatory and resistant mechanisms within the endogenous mineral regulatory network (133). In the pathogenesis of vascular calcification, dysregulation of endogenous mineral absorption pathways is manifested not only by enhanced pathological calcium deposition but also, more importantly, by impaired recalcification resolution, namely the defective capacity for active resorption of calcified deposits. Physiologically, osteoclast-mediated degradation of mineralized matrix maintains bone metabolic homeostasis, whereas this recalcification resolution mechanism is markedly constrained within the vascular microenvironment. Macrophages can polarize into osteoclast-like phenotypes and participate in extracellular matrix remodeling; nevertheless, vascular calcified lesions, particularly in the arterial media, are characterized by resistance to mineral resorption. Even in the presence of osteoclast-like cells, hydroxyapatite deposits cannot be efficiently cleared, suggesting that the calcified microenvironment may impose physical or biochemical barriers to cellular-mediated mineral resorption (134). In atherosclerotic plaques, macrophages are capable of eliminating early microcalcification foci and hydroxyapatite nucleation sites via phagocytosis. However, persistent inflammatory stimulation such as NLRP3 inflammasome-driven pyroptosis shifts macrophage phenotypes toward a pro-calcific profile. Once microcalcification progresses to macroscopic mineralization, the pathological process becomes largely irreversible (67). Emerging studies have validated that targeted modulation of macrophage function can partially restore mineral resorption capacity. For instance, enhancing chaperone-mediated autophagy (CMA) activity in osteoclast-like cells suppresses aberrant bone resorption (135). Specific exosomes such as Ancr/E7-EXO can reprogram perivascular Gli1⁺ cells to inhibit their osteogenic differentiation and alleviate vascular calcification (136). Additionally, *α*-ketoglutarate (AKG) has been verified to markedly attenuate calcium deposition in human and murine VSMCs (137). These findings highlight the therapeutic potential of rebalancing the vascular mineralization-resorption homeostasis. Future therapeutic strategies may focus on promoting the infiltration of functional osteoclast-like cells, regulating macrophage polarization such as inhibiting the CCL24 signaling axis, or indirectly modulating circulating pro-calcific factors using bone resorption inhibitors. Such approaches are expected to break through the limitations of current therapies that merely inhibit mineral deposition, providing novel intervention targets for CKD- and atherosclerosis-associated vascular calcification.

### Dysregulation of calcification inhibitors

The impairment of endogenous inhibitors of the extracellular matrix during the development of the mineralized milieu is a significant factor that promotes pathological calcification. Pyrophosphate (PPi), a naturally occurring extracellular mineralization inhibitor, exhibits markedly disrupted metabolic homeostasis under hyperglycemic conditions, such as diabetes mellitus. Hyperglycemia induces perturbations in enzyme activity, which directly reduces the pyrophosphate/phosphate (PPi/Pi) ratio and compromises its capacity to inhibit hydroxyapatite (HA) crystal deposition. Calcification assays in multiple diabetic models have consistently confirmed that this metabolic dysregulation enhances the susceptibility to vascular calcification (138). Meanwhile, hyperphosphatemia, a common hallmark of chronic kidney disease (CKD) and metabolic disorders, not only drives the osteogenic transdifferentiation of vascular smooth muscle cells (VSMCs) but also exacerbates functional PPi deficiency by disrupting its biosynthetic pathways (139). Fetuin-A, a hepatocyte-derived circulating mineralization inhibitor, blocks crystal growth by binding calcium and phosphate to form amorphous calciprotein particles (CPPs). However, under metabolic stress, its expression and function are substantially impaired: in the context of hyperglycemia and insulin resistance, casein kinase 2α (CK2α)-dependent phosphorylation of fetuin-A is decreased, facilitating its degradation via the autophagic pathway and resulting in a significant reduction in protein levels (140). A low fetuin-A concentration renders calcific deposits more prone to crystallization and pathological mineralization (141). Additionally, as an acute-phase reactant during inflammation, the dynamic changes of fetuin-A (e.g., peaking at 6 h followed by a decline in a traumatic brain injury model) reflect its complex role in the inflammation-mineralization axis (142), while the formation of crystalline CPPs further elicits oxidative stress and inflammatory cascades (143). Matrix Gla protein (MGP), a key vitamin K-dependent mineralization inhibitor, strictly relies on γ-carboxylation modification for its biological activity. Vitamin K deficiency, which is prevalent in hemodialysis patients, leads to the accumulation of undercarboxylated MGP (dp-ucMGP), which loses the ability to bind calcium ions and inhibit elastic fiber calcification. Meanwhile, accumulating evidence has confirmed that elevated circulating levels of dp-ucMGP are significantly correlated with arterial stiffness, cardiovascular events, and inflammation-associated pathological processes. These findings suggest that descarboxylated MGP not only impairs the endogenous inhibitory barrier against calcification, but also accelerates the progression of calcified lesions by exacerbating the inflammatory microenvironment in the vascular wall, such as through synergistic crosstalk with pro-inflammatory pathways including IL-1β (144–147). In diabetic and CKD models, MGP expression itself is also significantly downregulated (26), and its function is further regulated at the post-transcriptional level: the long non-coding RNA MALAT1 upregulates MGP expression by inhibiting miR-143-3p, and intervention of this axis can reverse the calcification process (148). Meanwhile, phosphorylation of MGP, which is dependent on the FAM20A-FAM20C kinase complex, is critical for its mineralization-inhibitory function (149). Notably, inflammatory signals (e.g., NF-κB activation) and oxidative stress [e.g., TXNIP-mediated reactive oxygen species (ROS) generation] not only directly promote VSMC calcification (150) but also form a vicious cycle by inhibiting the vitamin K cycle, interfering with MGP carboxylation, and accelerating fetuin-A degradation. Furthermore, the activity of PPi-metabolizing enzymes is regulated by inflammatory factors, which further impairs the protective effects of PPi (26).The deletion of the basement membrane protein Nidogen-2 was discovered to disturb the contractile phenotype of vascular smooth muscle cells (VSMC), promoting their development into osteoblast-like cells and impairing the homeostatic maintenance function of the extracellular matrix (ECM) (96). Likewise, the downregulation of calcium-binding protein expression (e.g., PALMD) can hinder the ECM's inhibitory influence on mineralization by disrupting signal pathways associated with matrix remodeling (e.g., TGF-*β*/Smad). Cartilage oligomeric matrix protein (COMP), a constituent of the vascular extracellular matrix, functions as an endogenous inhibitor of vascular calcification. A deficiency in COMP intensifies atherosclerotic calcification by promoting macrophage differentiation into atherosclerotic and osteogenic phenotypes through integrin β3 (151, 152). The malfunction of these inhibitors and the accumulation of pro-mineralizing components create a detrimental loop that ultimately results in irreversible vascular calcification.

### Extracellular vesicle-mediated mineral nucleation

Vascular calcification is an actively regulated pathological remodeling process, in which extracellular vesicles (EVs) serve as the pivotal nucleation core for mineralization. The mechanisms underlying EV biogenesis, cargo loading, and cell source-specific release collectively drive ectopic mineralization progression. Vascular smooth muscle cells (VSMCs) undergo osteogenic transdifferentiation upon stimulation with high phosphate, uremic serum, or oxidative stress, a process tightly regulated by mitochondrial dysfunction and endoplasmic reticulum stress. Concomitantly, VSMCs generate calcium- and phosphate-rich matrix vesicles (MVs) through an endosome system-dependent pathway. PIKFYVE, a lipid kinase that modulates endosomal maturation, is involved in EV biogenesis (153); meanwhile, the autophagy-lysosome pathway is aberrantly activated. Autophagosomes encapsulate mineral components and further differentiate into calcium-containing exosomes, which are then secreted into the extracellular matrix (ECM). Inhibition of the release of these EVs can significantly reduce calcium deposition (154). The concentrated calcium, phosphate, and specific proteins within EVs constitute the initial microenvironment for hydroxyapatite (HA) mineralization. Collagen fibers not only provide a structural scaffold for mineral deposition but also facilitate mineral nucleation by guiding EV aggregation in the pore regions (2, 155). Cargo loading of EVs is highly selective: exosomes secreted by endothelial cells under high phosphate or uremic conditions exhibit a significant upregulation of miR-670-3p, which promotes VSMC calcification by activating the Wnt/β-catenin signaling pathway (156). Exosomes derived from human umbilical vein endothelial cells (HUVECs) treated with high phosphate carry STAT1 protein, which also participates in this process (88). Following macrophage polarization, pro-inflammatory phenotypes secrete EVs containing pro-calcific microRNAs (e.g., miR-32-5p) or the long non-coding RNA MALAT1, whereas anti-inflammatory phenotypes typically exert a protective effect by secreting exosomes loaded with inhibitory molecules such as miR-204. However, anti-inflammatory macrophages may switch to a pro-calcific phenotype under metabolic disorders such as diabetes (148, 157, 158). Additionally, EVs derived from bone marrow mesenchymal stem cells can deliver the anti-osteogenic miR-133 to pathologically activated VSMCs, offering a novel strategy for therapeutic intervention (159). The functional specificity of EVs is determined by their cellular origin: VSMC-derived EVs, including apoptotic bodies, are the core nucleation sites for medial vascular calcification, and their release is regulated by the IKK2/NF-κB pathway. Inhibition of this pathway can enhance the mineralization capacity of EVs (160, 161). Under uremic or high-phosphate stimulation, endothelial cell-derived exosomes (e.g., HUVEC-Exos) are internalized by VSMCs to transmit pro-calcific signals, while pretreatment of endothelial cells with melatonin can alter the cargo content of their exosomes to inhibit calcification (156, 162). Macrophage-derived EVs bidirectionally regulate the calcification process through inflammatory factors, apoptotic bodies, and specific microRNAs, and their polarization state is profoundly influenced by the local microenvironment (157, 158). Notably, exosomes secreted by vascular adventitial fibroblasts under high-phosphate stimulation can also promote VSMC calcification (163), and EVs derived from hepatocytes associated with fatty liver disease have been found to be involved in the regulation of vascular calcification (164), highlighting a multi-cellular collaborative network in this process. In summary, through cell type-specific regulatory mechanisms of biogenesis, precise cargo sorting, and targeted intercellular delivery, EVs orchestrate a cascade reaction from micro-calcific foci to macroscopic mineralization in vascular calcification. In-depth dissection of these mechanisms is of great significance for the development of targeted anti-calcific therapies, such as engineered exosome delivery systems, and provides a solid theoretical basis for translational medicine.

### Enrichment of pro-mineralization matrix components

In vascular calcification, pro-mineralizing elements of the extracellular matrix (ECM) establish the foundational structure of the mineralized milieu by dynamic remodeling. In pathological conditions, the release of pro-mineralizing proteins (such as collagen type I, osteocalcin OCN, BMP-2, etc.) by osteoblast-like phenotypic cells primarily drives the remodeling of matrix components. Elastin serves as a fundamental matrix element in the calcification of blood vessel membranes, with its degradation products (e.g., exposed carboxylic acid groups) offering preferential sites for hydroxyapatite (HAP) deposition, particularly in pathological states like chronic kidney disease. In such conditions, the abnormal degradation of elastin markedly facilitates the deposition of calcium-phosphorus complexes through the activation of matrix metalloproteinases (e.g., MMP-3) (6, 165). In addition, elastin degradation products can act as damage-associated molecular patterns (DAMPs) to activate inflammatory signaling cascades, further amplifying the osteogenic phenotypic transition of VSMCs and accelerating the progression of vascular calcification (166). Type I collagen, a principal element of the extracellular matrix, serves as a structural scaffold for hydroxyapatite crystals and facilitates ectopic mineralization via direct interaction of its carboxyl terminus with apatite. However, its propensity for mineralization is inferior to that of elastin, a difference potentially attributable to the varying tissue distribution and modification states of collagen in physiological bone mineralization compared to pathological vascular calcification (167–169). Moreover, glycosaminoglycans like chondroitin sulphate (CS) can promote collagen intrafibrillar mineralization in the unbound state through electrostatic interactions, and their concentration-dependent influence further expedites the calcification process (170). Simultaneously, matrix metalloproteinases (MMPs) liberate pro-calcification fragments through the degradation of extracellular matrix structural proteins (e.g., elastin, collagen), while also activating pro-inflammatory and pro-fibrotic signal pathways, so establishing a positive feedback loop that expedites vascular wall mineralization (6, 171). Abnormal enrichment of certain bone-derived proteins facilitates calcification: stromal cell signal proteins (e.g., periosteal proteins) enhance the pro-calcification phenotypic transformation of vascular smooth muscle cells (VSMCs) through the integrin signal pathway, while promoting extracellular matrix (ECM) remodeling and inflammatory responses, thereby accelerating hydroxyapatite deposition (172); osteocalcin (OCN) induces calcification via the Wnt/β-catenin signal pathway and diminishes the production of pyrophosphate, a calcification inhibitor, by reducing glycolysis (51); and BMP-2 activates Smad1/5/8 signal in adjacent cells through an autocrine/paracrine mechanism, creating a “pro-mineralizing protein-signal amplification” positive feedback loop. The aforementioned mechanisms indicate that the augmentation of pro-mineralization matrix constituents arises not solely from an imbalance in calcium and phosphorus metabolism, but also from an active process involving alterations in extracellular matrix structure, aberrations in cellular signal, and a dynamic imbalance between mineralization inhibitors and promoters.

## Clinical interventions and translational dilemmas of vascular calcification

At present, clinical interventions for vascular calcification mainly rely on the management of underlying diseases, regulation of mineral metabolism, lipid-lowering and anti-inflammatory strategies, as well as interventional device therapy. Overall, there is still a lack of specific drugs capable of reversing established calcification. The translational landscape is characterized by abundant basic research findings but relatively lagging clinical implementation. Although basic research has gradually elucidated the key molecular pathways and regulatory targets underlying vascular calcification, emerging candidates including PDK4, PPARγ, PDZK1, TXNIP, Sirt7, DMY, and small extracellular vesicle miRNAs have displayed pronounced anti-calcification effects in preclinical studies. Nevertheless, most of these potential targets are still confined to cellular and animal models, with a lack of mechanism-oriented clinical validation.

Conventional interventions include the regulation of calcium-phosphorus metabolism, supplementation of vitamin K and magnesium preparations, administration of statins, SGLT2 inhibitors, bisphosphonates and anti-inflammatory agents. However, clinical trials of these therapies show prominent outcome heterogeneity and population-dependent therapeutic benefits (173). Statins can stabilize atherosclerotic plaques and moderately modulate mineralization progression, yet they fail to halt advanced calcification and may even exacerbate the mineral burden of the vascular wall (47). Vitamin K restores the endogenous anti-calcification barrier by promoting γ-carboxylation of matrix Gla protein, while magnesium preparations antagonize calcium-phosphorus toxicity and maintain vascular matrix homeostasis. Both exert definite anti-calcification effects in cellular and animal models, but their clinical efficacy is confounded by patients’ baseline nutritional status, renal function and inflammatory background. Large-sample randomized controlled trials have yielded inconsistent outcomes, accompanied by potential risks such as bone metabolic imbalance (21, 174, 175). Although bisphosphonates inhibit ectopic hydroxyapatite deposition, they tend to interfere with physiological bone remodeling, and long-term use may reduce bone mineral density and increase fracture risk. Novel hypoglycemic agents such as SGLT2 inhibitors, targeted anti-inflammatory drugs and ENPP1 replacement therapies have shown promising anti-calcification potential in preclinical studies. Nevertheless, most remain in Phase II/III clinical trials; only small cohort studies have observed delayed calcification progression, and high-level evidence is still lacking to support their widespread clinical application (31, 32).

Other interventions, including exercise, vitamin E-coated or high-flux dialysis membranes, anticoagulants, intradialytic sodium bicarbonate, SNF472, spironolactone, sotatercept, nicotinamide and activated charcoal, have yielded inconsistent results and lack high-quality clinical evidence (173, 176). In addition, targeted therapies for iron metabolic imbalance (177) and nanomedicine-based combination strategies (178) have demonstrated experimental potential but have not yet undergone clinical validation. Several natural compounds, such as melatonin (162), ursolic acid (179), emodin (180) and dihydromyricetin (181), have been reported to suppress vascular calcification-related signaling pathways, though none have completed clinical translation.

In terms of device intervention, tissue-engineered vascular grafts exhibit a lower long-term calcification degree than polytetrafluoroethylene grafts in both clinical investigations and animal models, suggesting that biomaterial optimization represents a promising future direction (182). From an interventional perspective, coronary rotational atherectomy, excimer laser ablation and intravascular shock wave lithotripsy can effectively treat severely calcified lesions and improve procedural success rates; however, these approaches are merely local mechanical interventions and cannot block the systemic pathological process of calcification. Overall, major limitations of current clinical trials include insufficient sample size, short follow-up duration, lack of standardized endpoints, and substantial inconsistency among published studies.

In addition, there are currently no effective drugs or specific reversal therapies for vascular calcification. Considering the remarkable differences in pathological mechanisms and clinical manifestations of vascular calcification occurring at different locations, targeted anti-calcification strategies need to be developed accordingly. Medial calcification frequently affects patients with diabetes, chronic kidney disease (CKD) and elderly individuals. It is deemed irreversible under physiological conditions owing to widespread calcium-phosphate deposition in the elastic fiber layer and the lack of osteoclast-mediated resorption mechanisms, and is strongly associated with vascular stiffness and increased risk of heart failure. Accordingly, early imaging identification should be emphasized to prevent misdiagnosis resulting from overlap with intimal calcification and optimize endovascular treatment regimens. In comparison, intimal calcification is mostly associated with atherosclerotic plaques, and its intervention places greater emphasis on regulating plaque stability, as extensive calcification may even exert plaque-stabilizing effects. Meanwhile, overlapping or ill-defined calcified lesions tend to cause interventional operation failure, so precise classification prior to clinical intervention is of great significance for individualized therapy (Table 1). As for universal intervention strategies, research priorities should focus on osteogenic transdifferentiation of vascular smooth muscle cells, oxidative stress and endoplasmic reticulum stress pathways. For instance, SGLT2 inhibitors exert beneficial effects on both types of calcification models by suppressing the TXNDC5/Runx2 signaling axis.

**Table 1:** Classification, core molecular mechanisms and potential targeted therapies of vascular calcification (VC).

VC Subtype	Predisposing Disease & Vascular Location	Dominant Cellular Event	Core Molecular Signaling & Regulatory Mechanisms	Key Upstream Risk Factors	Potential Targeted Therapeutic Strategies
Medial Vascular Calcification	Chronic kidney disease (CKD) Diabetes mellitus Elderly arterial media	VSMC osteogenic transdifferentiation (dominant), EndoMT & Macrophage polarization (auxiliary amplification)	1. Wnt/*β*-catenin, BMP/SMAD, PI3 K/Akt signaling activation 2. RUNX2/MSX2/SOX9/OSX transcriptional network upregulation 3. Hyperphosphatemia-induced TNAP elevation, PPi/phosphorus ratio imbalance 4. Mitochondrial dysfunction, mtROS overproduction 5. Ferroptosis/cuproptosis-mediated mineral vesicle release	Calcium-phosphate imbalance Glucolipotoxicity Mitochondrial damage Hypertension-induced hemodynamic abnormality	1. Target VSMC phenotypic switching: PDK4, PPAR*γ* agonists 2. Regulate mineral metabolism: Magnesium/zinc supplementation, vitamin K to restore MGP carboxylation 3. Mitochondria-targeted antioxidants (MitoQ) 4. Inhibit Wnt/BMP pathway activation 5. Block ferroptosis/cuproptosis signaling
Atherosclerotic Intimal Calcification	Atherosclerosis Arterial intima & plaque	EndoMT initiation,Heterogeneous macrophage polarization (SPP1⁺/LYVE-1⁺ subsets),VSMC phenotypic switching (downstream effector)	1. NLRP3 inflammasome, NF-*κ*B inflammatory cascade 2. Ox-LDL-induced ER stress (PERK/eIF2*α*/CHOP axis) 3. Gut microbiota-SCFA metabolic regulation 4. EVs-mediated intercellular mineral signal transmission 5. Macrophage osteoclast-like differentiation & mineral resorption imbalance	Lipotoxicity (ox-LDL) Turbulent shear stress at arterial bifurcations Chronic inflammation Gut microbiota dysbiosis	1. Stabilize plaque via moderate calcification: Statins 2. Regulate macrophage polarization: H₂S, PPARγ agonists, TLR4 inhibitors 3. Target EndoMT: GSK3β inhibitor (SB216763), Cinacalcet 4. Modulate gut microbiota to regulate SCFA metabolism 5. Engineered exosome intervention
Diabetic Combined VC	Type 2 diabetes Mixed arterial media + intima	Co-activation of VSMC osteogenic transdifferentiation, EndoMT and pro-inflammatory macrophage polarization	1. AGEs-RAGE axis overactivation 2. Glycolysis reprogramming & BNIP3-related mitophagy inhibition 3. ENPP1 activity suppression leading to PPi deficiency 4. Epigenetic reprogramming (m6A, lncRNA H19) 5. Pyroptosis-driven mineralization initiation	Hyperglycemia Insulin resistance Lipid metabolic disorder	1. SGLT2 inhibitors, GLP-1 receptor agonists 2. Target AGEs-RAGE signaling pathway 3. Epigenetic intervention (target m6A modification, circRNA/miRNA axis) 4. Inhibit NLRP3 inflammasome and pyroptosis

## Conclusions and perspectives

Vascular calcification represents a common pathological endpoint of multiple cardiovascular diseases, and its pathogenesis involves complex regulation of multicellular crosstalk and multiple signaling pathways. This review systematically summarizes the core driving role of vascular smooth muscle cell (VSMC) phenotypic switching in vascular calcification. VSMCs remodel the extracellular matrix (ECM) and activate the osteogenic differentiation network, ultimately leading to the formation of irreversible mineralized lesions.

Although current basic research has gradually clarified the key molecular pathways and regulatory targets underlying vascular calcification, the translation from mechanistic investigations to precise clinical intervention still faces multiple bottlenecks and practical challenges. First, the clinical benefits of conventional anti-calcification therapies remain highly controversial with substantial heterogeneity in trial outcomes. Magnesium supplementation exerts definite anti-calcification effects in cellular and animal models by antagonizing calcium-phosphorus toxicity, maintaining matrix integrity, and inhibiting VSMC osteogenic transdifferentiation. However, large-sample clinical trials have yielded inconsistent therapeutic efficacy, with no obvious benefits observed in certain populations. Moreover, long-term magnesium supplementation may suppress physiological bone mineralization and increase the risks of osteomalacia and fracture. Vitamin K restores the endogenous anti-calcification barrier by promoting MGP γ-carboxylation; nevertheless, clinical studies on vitamin K supplementation in dialysis patients have drawn conflicting conclusions, which are markedly confounded by patients’ baseline nutritional status, inflammatory levels, and the severity of renal impairment. Bisphosphonates can inhibit ectopic hydroxyapatite deposition but tend to disrupt the normal bone remodeling balance, causing off-target effects such as suppressed bone metabolism and abnormal reduction in bone mineral density in some populations. Existing reviews only briefly mention the potential value of the above therapies, but fail to thoroughly analyze the core reasons for the discrepancy between preclinical efficacy and clinical trial outcomes, including pathological background differences between preclinical models and real patients, variations in drug dosage and administration timing, interference from combined medication, and bidirectional imbalance of the bone-vascular metabolic axis.

Second, targeted intervention for vascular calcification is hampered by severe off-target effects and paradoxical therapeutic windows. A typical bidirectional regulatory window exists in atherosclerotic calcification: moderate mineralization in the early stage stabilizes vulnerable plaques and reduces the risk of rupture, whereas excessive calcification in the middle and advanced stages induces vascular stiffness and luminal stenosis, increasing the difficulty of interventional therapy. Statins serve as a typical example; they lower blood lipids and promote moderate plaque mineralization to achieve short-term plaque stabilization, yet long-term administration continuously induces VSMC osteogenic phenotypic switching, aggravates the burden of advanced vascular calcification, and meanwhile disturbs bone metabolic homeostasis. Although various inhibitors of osteogenic signaling pathways can block the progression of vascular calcification (VC), they inevitably interfere with physiological bone development and bone remodeling, making it difficult to achieve the therapeutic goal of vascular targeted protection without disrupting normal bone mineralization. This also constitutes a major obstacle to the clinical translation of current small-molecule drugs.

More importantly, there is a prominent translational gap between preclinical animal models and genuine human vascular calcification, which has become a core bottleneck restricting the clinical application of basic research targets. The first issue lies in patient heterogeneity: patients with clinical VC often suffer from multiple comorbidities such as chronic kidney disease, diabetes, advanced age, hypertension, and lipid disorders. There are substantial individual differences in genetic background, gut microbiota, baseline inflammation, and mineral metabolic reserve. In contrast, most basic studies adopt inbred animal models with a single etiological factor, which fail to recapitulate the complex comorbid conditions in real clinical settings, resulting in poor reproducibility of target efficacy in clinical trials. The second concern is the irreversibility of advanced calcification: most preclinical experiments focus on the early microcalcification stage, in which phenotypic switching and matrix remodeling are still amenable to reversible regulation. However, most clinically admitted patients present with moderate to advanced macroscopic calcification, characterized by massive deposition of hydroxyapatite crystals, permanent destruction of extracellular matrix architecture, and solidified multicellular pro-calcification positive feedback loops. Under such circumstances, targeting a single molecular pathway alone is hardly sufficient to reverse the pathological process. Third, off-target disturbance to the bone-vascular metabolism axis is almost unavoidable. Vascular calcification and physiological bone mineralization share core osteogenic signaling pathways including RUNX2, BMP, and Wnt/β-catenin. Current targets lack vascular tissue specificity; interventions against VC readily perturb bone metabolic homeostasis, leading to adverse reactions such as bone loss and elevated fracture risk, thereby limiting long-term clinical application.

In summary, substantial research gaps remain regarding the spatiotemporal regulatory mechanisms of metabolic disorders, fine epigenetic regulation, and the crosstalk between novel types of cell death and calcification. Future studies should further elucidate the mechanisms underlying the heterogeneous efficacy of classical therapies such as magnesium and vitamin K, and optimize applicable populations and administration regimens. It is imperative to develop multi-target drugs with anti-inflammatory, anti-oxidant, and anti-osteogenic transdifferentiation properties, establish clinically relevant complex-etiology animal models and organoid systems to narrow the translational gap, screen novel calcification biomarkers via multi-omics approaches, and develop targeted delivery systems such as nanocarriers and engineered exosomes. These strategies will enable precise vascular drug administration while avoiding off-target impacts on bone metabolism, and ultimately facilitate early warning and precise clinical intervention of vascular calcification.
