# Supplementary material for: Mechanisms of vascular calcification: cellular phenotype switching drives matrix remodeling and mineralized microenvironment formation
Source: Front Cardiovasc Med. 2026 Jun 18;13:1864140. doi: 10.3389/fcvm.2026.1864140 (PMC13322825; doi:10.3389/fcvm.2026.1864140)
Supplement: Supplementary file 1 [file Table1.docx]

| **Abbreviation** | **Full Name** |
| --- | --- |
| α-SMA | α-smooth muscle actin |
| AGEs | Advanced glycation end products |
| ALP | Alkaline phosphatase |
| AS | Atherosclerosis |
| Arg-1 | Arginase-1 |
| ATF4 | Activating transcription factor 4 |
| BNIP3 | Bcl-2/adenovirus E1B 19 kDa interacting protein 3 |
| BMP2 | Bone morphogenetic protein 2 |
| Ca²⁺ | Calcium ion |
| CaSR | Calcium-sensing receptor |
| C5a | Component 5a |
| C5aR1 | Component 5a receptor 1 |
| CD31 | Cluster of differentiation 31 |
| CDK1 | Cyclin-dependent kinase 1 |
| CHOP | C/EBP homologous protein |
| CKD | Chronic kidney disease |
| Col Ⅰ | Collagen type I |
| COMP | Cartilage oligomeric matrix protein |
| CPPs | Calciprotein particles |
| Celastrol | Celastrol |
| circACTA2 | Circular RNA ACTA2 |
| circRNAs | Circular RNAs |
| circ_0008362 | Circular RNA 0008362 |
| ECM | Extracellular matrix |
| ENPP1 | Ectonucleotide pyrophosphatase/phosphodiesterase 1 |
| EndoMT | Endothelial-mesenchymal transition |
| ERK1/2 | Extracellular regulated protein kinases 1 and 2 |
| ETV2 | Ets variant 2 |
| EPO | Erythropoietin |
| eIF2α | Eukaryotic initiation factor 2α |
| Exendin-4 | Exendin-4 |
| FGF23 | Fibroblast growth factor 23 |
| FAK | Focal adhesion kinase |
| Fetuin-A | α2-Heremans-Schmid glycoprotein |
| FDX1 | Ferredoxin 1 |
| GLP-1 | Glucagon-like peptide-1 |
| GNPTAB | Glucosamine (N-acetyl)-6-sulfatase |
| GRP | Gla-rich protein |
| GSK3β | Glycogen synthase kinase 3β |
| GSDMD | Gasdermin D |
| GPX4 | Glutathione peroxidase 4 |
| H19 | Long chain non-coding RNA H19 |
| H₂S | Hydrogen sulfide |
| HDAC | Histone deacetylase |
| HDL-C | High-density lipoprotein cholesterol |
| H3K14la | Lactylation of histone H3 at lysine 14 |
| H3K18la | Lactylation of histone H3 at lysine 18 |
| H3K9me3 | Trimethylation of histone H3 at lysine 9 |
| IL-10 | Interleukin-10 |
| IL-1β | Interleukin-1β |
| IL-6 | Interleukin-6 |
| IRE1 | Inositol-requiring enzyme 1 |
| JAK-STAT | Janus kinase-signal transducer and activator of transcription |
| KLF2 | Kruppel-like factor 2 |
| Klotho | Klotho protein |
| LDL-C | Low-density lipoprotein cholesterol |
| Lactate | Lactate |
| MAPK | Mitogen-activated protein kinase |
| MAPKs | Mitogen-activated protein kinases |
| MACE | Major adverse cardiovascular events |
| MGP | Matrix Gla protein |
| MMPs | Matrix metalloproteinases |
| Mg²⁺ | Magnesium ion |
| MitoQ | Mitochondria-targeted coenzyme Q |
| miR-155 | MicroRNA-155 |
| miR-1251-5p | MicroRNA-1251-5p |
| mitophagy | Mitophagy |
| M1 | Classically activated macrophages |
| M1-EVs | M1 macrophage-derived extracellular vesicles |
| M2 | Alternatively activated macrophages |
| mtROS | Mitochondrial reactive oxygen species |
| MVs | Matrix vesicles |
| NADPH | Nicotinamide adenine dinucleotide phosphate |
| NCX1 | Sodium-calcium exchanger 1 |
| NF-κB | Nuclear factor-κB |
| NFATc1 | Nuclear factor of activated T cells cytoplasmic 1 |
| NLRP3 | NOD-like receptor pyrin domain-containing 3 |
| Nox5 | NADPH oxidase 5 |
| Nrf-2 | Nuclear factor erythroid 2-related factor 2 |
| NR4A3 | Nuclear receptor subfamily 4 group A member 3 |
| OCN | Osteocalcin |
| OPG | Osteoprotegerin |
| OPN | Osteopontin |
| Ost-MVs | Osteoblast-derived matrix vesicles |
| ox-LDL | Oxidized low-density lipoprotein |
| Palmitic acid | Hexadecanoic acid |
| PALMD | Palmdelphin |
| PDK4 | Pyruvate dehydrogenase kinase 4 |
| PERK | Protein kinase R-like endoplasmic reticulum kinase |
| PI3K/Akt | Phosphatidylinositol 3-kinase/Protein kinase B |
| Pi | Inorganic phosphate |
| PiT-1/2 | Sodium-dependent phosphate transporters 1 and 2 |
| PPARγ | Peroxisome proliferator-activated receptor γ |
| PPi | Pyrophosphate |
| PTCA | Percutaneous transluminal angioplasty |
| PTH | Parathyroid hormone |
| PUFAs | Polyunsaturated fatty acids |
| RAGE | Receptor for advanced glycation end products |
| ROS | Reactive oxygen species |
| RUNX2 | Runt-related transcription factor 2 |
| SASP | Senescence-associated secretory phenotype |
| SCFAs | Short-chain fatty acids |
| SGLT2 | Sodium-glucose cotransporter 2 |
| SIRT1 | Sirtuin 1 |
| SIRT3 | Sirtuin 3 |
| SIRT7 | Sirtuin 7 |
| SMAD | Sma and Mad related proteins |
| SM22α | Smooth muscle protein 22 alpha |
| SOX9 | SRY-box transcription factor 9 |
| SPP1 | Secreted phosphoprotein 1 |
| SR-A/SR-B1 | Scavenger receptor A/scavenger receptor B1 |
| STAT6 | Signal transducer and activator of transcription 6 |
| STING1 | Stimulator of interferon genes 1 |
| TGF-β | Transforming growth factor-β |
| TGF-β1 | Transforming growth factor-β1 |
| TLR4 | Toll-like receptor 4 |
| TNF-α | Tumor necrosis factor-α |
| TNAP | Tissue non-specific alkaline phosphatase |
| TRAP | Tartrate-resistant acid phosphatase |
| VC | Vascular calcification |
| VE-cadherin | Vascular endothelial cadherin |
| Vimentin | Vimentin |
| VSMC-MVs | Vascular smooth muscle cell-derived matrix vesicles |
| VSMCs | Vascular smooth muscle cells |
| Wnt/β-catenin | Wingless-type MMTV integration site family/β-catenin |
| XBP1 | X-box binding protein 1 |
| Zn²⁺ | Zinc ion |
| ω-3 PUFAs | Omega-3 polyunsaturated fatty acids |
| m⁶A | N6-methyladenosine |
| METTL3 | Methyltransferase-like 3 |
| SOD2 | Superoxide dismutase 2 |
| ucMGP | Undercarboxylated matrix Gla protein |
